# Subacute Blindness Revealing an Autoimmune Glial Fibrillary Acidic Protein Astrocytopathy

**DOI:** 10.7759/cureus.17588

**Published:** 2021-08-31

**Authors:** Fatima Zahra Mabrouki, Faiza Aziouaz, Rachid Sekhsoukh, Mebrouk Yassine

**Affiliations:** 1 Ophthalmology, Mohammed VI University Hospital of Oujda/Faculty of Medicine and Pharmacy of Oujda, Oujda, MAR; 2 Neurology, Mohammed VI University Hospital of Oujda/Faculty of Medicine and Pharmacy of Oujda, Oujda, MAR

**Keywords:** gfap, encephalitis, autoimmune, bilateral optic neuritis, blindness

## Abstract

Autoimmune glial fibrillary acidic protein (GFAP) astrocytopathy is an autoimmune disease of the central nervous system characterized by positive GFAP autoantibody. The most common are encephalitis, meningoencephalitis or meningoencephalomyelitis. Antibodies in cerebrospinal fluid (CSF) against GFAP are biomarkers and expressed in most cases with autoimmune GFAP astrocytopathy. Diagnosis by biopsy is not common practice and has been rarely performed in the literature. This is the particularity of our reported case of autoimmune GFAP astrocytopathy presented as opticopyramidal syndrome, all paraclinical investigations were normal, only the biopsy allowed the diagnosis.

## Introduction

Autoimmune glial fibrillary acidic protein (GFAP) astrocytopathy is an autoimmune disease recently described by the central nervous system (CNS) [1]. The common clinical features, including fever, headache, encephalopathy, involuntary movement, myelitis, and visual abnormalities, have been reported [2]. Antibodies in cerebrospinal fluid (CSF) against GFAP are biomarkers and expressed in most cases with autoimmune GFAP astrocytopathy [3]. Diagnosis by biopsy is not common practice. It has been performed rarely in the literature [4]. Herein, we reported a case of autoimmune GFAP astrocytopathy presented as opticopyramidal syndrome diagnosed by biopsy.

## Case presentation

**Figure 1:**
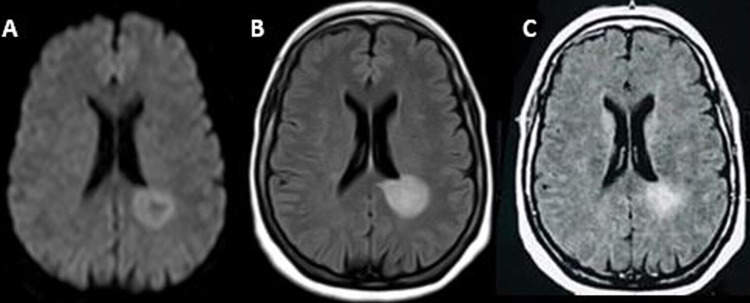
Brain magnetic resonance imaging (1.5 Tesla) showed two pseudo-nodular lesions of the peri-ventricular white matter, hyperintense in T2 and T2 flair, isointense in T1, with diffusion restriction, associated with other small lesions of the white matter on the left frontal level, right insular and bilateral occipital, enhanced after injection of gadolinium (A) Diffusion axial view; (B) T2 fluid-attenuated inversion recovery (FLAIR) axial view; (C) T1 axial view with gadolinium.

A 49-year-old female, without any medical history, presented at our hospital on August 8, 2020, with heaviness in all four limbs, accompanied with a profound drop in visual acuity, without pain, first in the left eye and then in the right eye, without any notion of headache or diplopia. Motor system examination revealed normal bulk in all four limbs. There was hypotonia in both lower limbs. Muscular testing was grade 0/5 in both lower limbs and grade 5/5 in both upper limbs. There was no involuntary movement. Plantar reflexes were bilaterally mute. All modalities of sensation were preserved on admission to the neurology department. His extra neurological examination was normal. Her neurological examination, revealed a tetraparesis with power grade 1/5 in the right hemibody proximo-distal, 3/5 in the left hemibody proximo-distal. There was hypotonia in four members and bilaterally Babinski. Her ophthalmologic examination found visual acuity where only the movements of the fingers were visible at the two eyes, direct and consensual photo-motor reflex abolished bilaterally and bilateral stage II papillary edema. No abnormal founding in other cranial nerves was observed, cognitive function and neck rigidity were normal. Clinical examination of other systems did not reveal any abnormalities. A brain and a spinal cord MRI were performed, which objectified two nodular lesions of the peri-ventricular white matter, bilateral, measuring 22 mm on the right one and 33 mm on the left one, associated with other small lesions of the white matter on the left frontal level, right insular and bilateral occipital, enhanced after injection of gadolinium, suggesting an inflammatory (multiple sclerosis) or infectious origin (acute encephalomyelitis) (Figures 1A-1C). This clinical presentation, as well as the radiological findings described previously, makes it possible to evoke the following etiologies: inflammatory origin (pseudotumoral MS, ADEM, NMO), infectious origin (neuro-HIV, toxoplasmosis) and tumor origin (lymphoma, metastases).

**Figure 2:**
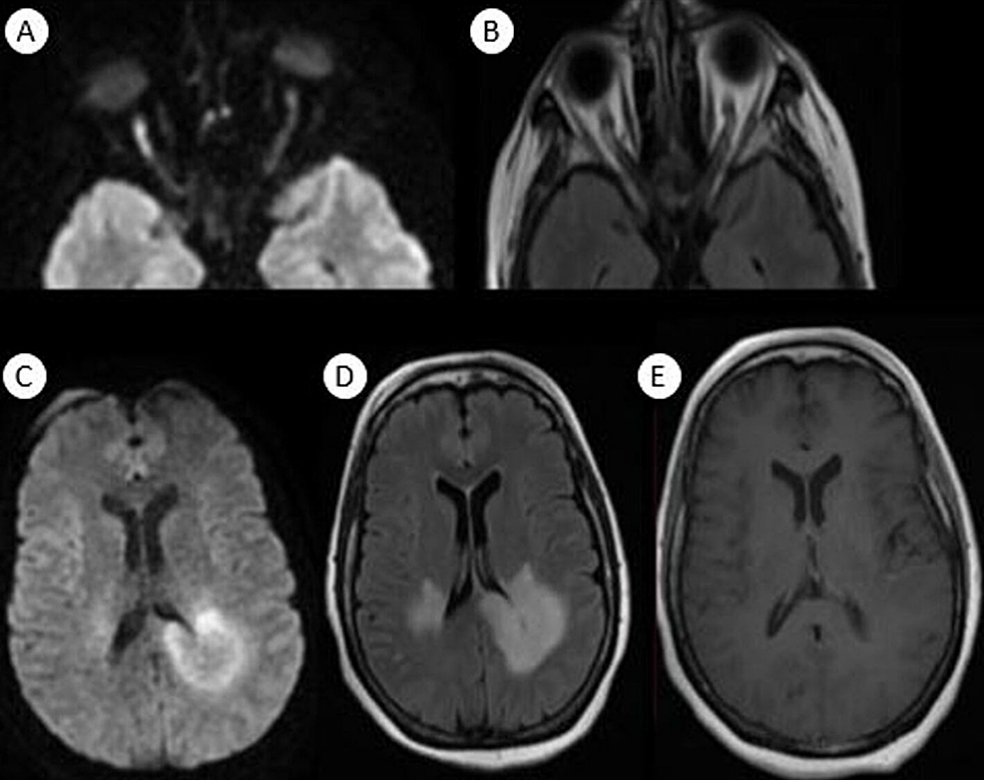
Orbital and cerebral magnetic resonance imaging after four weeks showed extension of previous lesions with involvement of the corpus callosum with diffusion restriction and peripheral enhancement, and appearance of bilateral optic neuritis more obvious on the right optic nerve than on the left one (A) Axial diffusion view of orbital magnetic resonance imaging; (B) axial T2 fluid-attenuated inversion recovery (FLAIR) view of orbital magnetic resonance imaging; (C) axial diffusion view of cerebral magnetic resonance imaging; (D) axial T2 FLAIR view of cerebral magnetic resonance imaging; (E) axial T1 Gado of cerebral magnetic resonance imaging.

Biochemical and cytological studies of the CSF showed an elevated protein count (0.7g/L) and normal glucose without pleocytosis. No oligoclonal band was detected in the CSF analysis. She tested negative for serum aquaporin-4 IgG and MOG-IgG (myelin oligodendrocyte glycoprotein antibodies). Serology for HIV, treponema pallidum hemagglutination (TPHA), venereal disease research laboratory (VDRL), hepatitis B and C were all negative. Laboratory results such as copper, B12, B9 levels were checked and were within the reference range. The patient reported no family history of cancer, and computed tomography (CT) imaging of the thorax, abdomen and pelvis was done to assess possible malignancy, which was negative. The patient received bolus methylprednisolone (1g per day for 10 days), an immunoglobulin cure (0.4g/kg for five days) and motor physiotherapy sessions, with prevention of thromboembolic complications. No clinical improvement was observed. A brain MRI was performed after four weeks, and showed multiple lesions of the supratentorial white matter with diffusion restriction and peripheral enhancement, with an appearance of bilateral optic neuritis (Figures 2A-2E).

Given the clinical and radiological non-improvement despite a well-adapted treatment, a stereotaxic brain biopsy was performed (after a brain scan without and with injection requested by the neurosurgeons) showing an inflammatory aspect with anti-GFAP antibodies. The patient benefited from a second immunoglobulin dose, methylprednisolone bolus 1g (five days), Cyclophosphamide 1g per day (three days) and seven sessions of plasmapheresis. The evolution was marked by the partial improvement of the deficit but persistence of blindness.

## Discussion

Autoimmune GFAP astrocytopathy is an inflammatory CNS disorder. It may affect any anatomic region, rostrocaudally, from the optic nerve to the spinal cord [1]. The first case was reported in 2016 revealed by meningoencephalomyelitis [2].

It usually occurs in patients after 40 years old (median age of onset: 44-50) and affects men and women equally [1,3]. Despite female predominance among paraneoplastic teratoma-accompanied cases [1]. Children account for approximately 10% [3].

The most common presentations of autoimmune GFAP astrocytopathy were fever, headache, and meningoencephalitis symptoms presented 1 or 2 weeks later [1,4]. The usual neurological findings during hospitalization included meningeal signs and consciousness disturbance in the majority of patients [1,2,4]. However, other manifestations were previously reported as rare neurological accompaniments of autoimmune GFAP astrocytopathy such as movement disorders (tremor, myoclonus and ataxia) and autonomic dysfunction (mainly urinary dysfunction) [1,4].

Almost all patients have inflammatory CSF with lymphocyte-predominant elevation in white blood cells and elevated protein. Oligoclonal bands and elevated IgG can be founded [2,4,5]. The most clinically sensitive and specific diagnostic biomarker of this disorder is the detection of autoantibodies in CSF against GFAP, which is the predominant intermediate filament protein in adult astrocytes [2,5].

On brain MRI, the majority of abnormalities are on T2- weighted and FLAIR sequences. The most common abnormal hyperintensity lesions were in the basal ganglia, followed by the thalamus [4,6]. Some authors suggest that the bilateral hyperintensities of the posterior part of the thalamus are characteristic findings of autoimmune GFAP astrocytopathy [4,5]. It has been reported that brain linear perivascular radial gadoinium enhancement patterns are a hallmark of autoimmune GFAP astrocytopathy [2,4]. Lesions at the corpus callosum level on brain MRI, as in our case report, were reported in a single case of a 17-year-old girl with epileptic seizures and recurrent optic neuritis [6].

Currently, the pathophysiological mechanism of GFAP astrocytopathy is not clarified. a pathological study on GFAP astrocytopathy was performed in only five cases in the literature, our case report was the sixth [5,7]. Pathological biopsy showed that CD138+ plasma cells were present in the brain lesions of the patients [8-11]. This suggested that autoantibodies were synthesized in the brain, explaining why antibodies in the CSF are higher than in the peripheral blood. Furthermore, the continuous secretion of antibodies in the brain might affect the therapeutic effect. Previous cases reported in the literature showed that GFAP astrocytopathy might be secondary to other diseases [9].

GFAP astrocytopathy is often linked to other pathologies such as neoplastic disease [5,8]. About 25% of the patients were found to have tumors, among which, 75% were teratomas, and 20% of the patients had autoimmune diseases, including type 1 diabetes and rheumatoid arthritis [7,8].

Treatment of acute GFAP astrocytopathy includes high-dose corticosteroids, immunoglobulins, and plasma exchange [7,8]. Recent studies have confirmed its efficacy in shortening time recovery [6,9,10]. For recurrent forms, other therapies have proven their effectiveness such as mycophenolate mofetil, azathioprine, rituximab and cyclophosphamide [5,8,9].

## Conclusions

The pathophysiological mechanisms and the triggers of autoimmunity in GFAP still remain obscure; it seems that searching for anti-GFAP antibodies will become essential in the presence of any suggestive sign and the absence of a well-determined etiology. Diagnosis by biopsy is not common practice and has only been performed in rare cases in the literature. It was done in our context in the absence of a diagnostic orientation. The prognosis depends on the speed of treatment and the severity of the clinical signs.
